# Impact of Maxillary Palatal Expansion on Airway Dimensions and Sleep-Disordered Breathing

**DOI:** 10.3390/dj14010023

**Published:** 2026-01-04

**Authors:** Eileen Shah, Val Joseph Cheever, Veronica Lexa Marr

**Affiliations:** College of Dental Medicine, Roseman University of Health Sciences, 10894 S. River Front Parkway, South Jordan, UT 84095, USA; jcheever@roseman.edu (V.J.C.); vmarr207@student.roseman.edu (V.L.M.)

**Keywords:** obstructive sleep apnea, respiratory function, maxillary palatal expansion, rapid maxillary expansion, mini-implant assisted rapid palatal expansion, airway

## Abstract

Obstructive sleep apnea (OSA) is a prevalent disorder characterized by repeated upper airway collapse during sleep, significantly impacting quality of life. Orthodontists are increasingly recognized for their role in screening and managing anatomical factors contributing to airway obstruction. One such intervention is rapid maxillary expansion (RME), originally developed to address transverse maxillary deficiencies but now also studied for its influence on nasal and oropharyngeal airway dimensions. This literature review evaluates the effects of maxillary palatal expansion on airway volume and respiratory function. Evidence consistently shows increases in nasal cavity volume and reductions in nasal airway resistance, particularly in patients treated before the peak of skeletal growth. Some studies reported improvements in sleep outcomes and enhanced oxygen saturation following MARPE in adults with OSA. Results regarding changes in oropharyngeal volume were more variable, with several studies showing significant expansion. Factors influencing outcomes include patient age, skeletal maturity, appliance type, and aging modality. Hybrid and bone-borne expanders generally demonstrated greater skeletal effects compared to tooth-borne devices, though statistical significance was not always reached. While MARPE has shown promising results in non-obese adults with OSA, long-term stability of airway improvements and translation into consistent functional respiratory benefits remain uncertain. Overall, maxillary expansion demonstrates measurable skeletal and airway changes, with the greatest respiratory improvements in growing patients and selected adult populations. Incorporating patient-reported outcomes and standardized polysomnographic measures in future trials will be critical to determine whether these structural gains consistently translate into durable improvements in sleep-disordered breathing and quality of life.

## 1. Introduction

Sleep is a critical component of the overall quality of life. During normal sleep, air flows freely through the upper airway down the respiratory tract and into the lungs. However, the most prevalent reason for airway obstruction is due to soft tissues in the back of the throat that collapse, blocking airflow [1]. Repeated episodes of upper airway collapse and obstruction during sleep are key components of a condition known as obstructive sleep apnea (OSA) [2,3]. Patients who present with hypertrophic adenoids and tonsils can also lead to an obstructed airway, mouth breathing, and OSA, negatively affecting overall well-being and life quality [4].

Understanding the clinical components that suggest the presence of OSA or other airway-related conditions is essential in diagnosis. It is becoming increasingly important to incorporate a multidisciplinary model for managing OSA and airway-related conditions [1]. The position of an orthodontist is extremely valuable in identifying those at risk of developing sleep apnea.

Although obstructive sleep apnea must be diagnosed by a physician, orthodontists can play an impactful role in the screening process. An orthodontist can help contribute to the management of sleep apnea to optimize patient care [1]. Commonly contributing abnormalities that can predispose a patient to OSA include “mandibular deficiency, inferiorly placed hyoid bone relative to the mandibular plane, narrowed posterior air space, greater flexion of the cranial base, and elongation of the soft palate” [5]. Studies have shown that specific treatment options utilizing appliances for maxillary expansion or mandibular advancement can be effective in the management and treatment of certain airway conditions in children and young adolescents [1,6].

Maxillary palatal expansion (MPE) is an established treatment in orthodontic practice for transverse maxillary deficiencies. Originally used to correct imbalances within the occlusion, improve facial harmony and nasal airflow, its role has evolved to include respiratory function [4]. 

Given the maxilla’s essential role in shaping the upper airway, researchers continue to investigate how RME can influence nasal resistance, total airway volume, and other conditions relating to airway obstruction [4]. A constricted maxillary arch is frequently paired with compromised nasal airflow [7]. Due to the close anatomical proximity between the maxilla and the nasal cavity, communication between the two is extremely important for patients’ breathing [7]. Because the maxillary bones form 50% or more of the nasal cavity, the nasal airway contributes to about half of the total airway resistance [8]. Consequently, individuals with maxillary constriction can exhibit various symptoms that range from nasal obstruction to mouth breathing, compromising the amount of oxygen they intake.

The therapeutic appeal of maxillary expansion lies in its ability to widen the transverse dimension of the maxilla, thereby expanding the nasal passages and, in turn, reducing airway resistance [9]. Advanced imaging modalities, including cone-beam computed tomography (CBCT) and acoustic rhinometry (AR), have allowed researchers to quantify these changes with more accuracy and precision [10]. More recently, RME has been associated with changes in upper airway dimensions. However, the extent of these changes, the long-term effectiveness, and the relationship between airway dimensional changes and breathing capacity remain controversial, particularly regarding the oropharyngeal region [11].

The aim of the literature review is to examine current clinical research and results of RME and its relationship to the airway. To analyze RME techniques and their impact on airway size and performance.

## 2. Materials and Methods

### 2.1. Search Strategy

A narrative review was conducted to evaluate the impact of maxillary palatal expansion on obstructive sleep apnea. A literature search was conducted using databases such as PubMed, Google Scholar, Cochrane Library, and Science Direct Health Sciences Edition. The keywords that were included were “maxillary palatal expansion,” “rapid maxillary expansion,” “nasal airway resistance,” “mini-implant assisted rapid palatal expansion,” “airway”, and “obstructive sleep apnea (OSA).” The dates of the publications that were reviewed ranged to best represent both a historical and current view in the existing literature. The initial search across databases yielded 64 articles. Of note, 15 duplicates were removed, and the remaining 49 records were screened by 3 independent reviewers. Following this, 10 reports were not retrieved, and the remaining 39 reports were assessed by reviewers for eligibility. Of these remaining records, 12 were focused on either surgical intervention or mandibular advancement options or did not evaluate a type of maxillary palatal expansion with implications for OSA. 3 additional articles were non-English texts and were subsequently removed. Consequently, 25 remaining studies met all inclusion criteria.

### 2.2. Inclusion and Exclusion Criteria

Included studies evaluated the effects of maxillary expansion on nasal or oropharyngeal airway volume. Selected studies utilized imaging techniques including cone-beam computed tomography (CBCT), acoustic rhinometry (AR), and polysomnography. The included studies examined the impact of different RME devices, including tooth-borne, bone-borne, and hybrid expanders. In terms of participants, human subjects across pediatric, adolescent, or adult populations. Inclusion criteria for this review consisted of peer-reviewed, English-language studies. Exclusion criteria included articles focused exclusively on dentoalveolar outcomes without airway evaluation. Additionally, excluded publications relied on outdated or unverified measurement techniques or lacked sufficient data. Studies that focused on surgical maxillary expansion, mandibular advancement devices, and evaluation of only skeletal and behavioral trends without mentioning maxillary intervention were not included.

## 3. Results

A total of 25 studies met the inclusion criteria for this review, including patients across pediatric, adolescent, and adult populations. The sample sizes of the included studies ranged from small prospective cohorts of fewer than 20 patients to randomized controlled trials with more than 100 participants. Studies evaluated airway dimensional changes and assessed functional respiratory outcomes such as the apnea-hypopnea index (AHI) and oxygen saturation. The majority of research focused on traditional tooth-borne RME in growing patients, while several other studies examined mini-implant-assisted rapid palatal expansion (MARPE) in adults. These studies form clinical conclusions regarding the effects of maxillary expansion on nasal airway volume, oropharyngeal dimensions, and sleep-disordered breathing.

Ref. [2] examined the effectiveness of mini-implant-assisted rapid palatal expansion (MARPE) in adults diagnosed with obstructive sleep apnea (OSA) [2]. Their multicenter trial documented a significant 65.3% reduction in the apnea-hypopnea index (AHI) as well as improved oxygen saturation and sleep quality. Cantarella et al. [12] demonstrated that MARPE induces separation not only in the midpalatal suture but also in the pterygopalatine suture complex, as confirmed through three-dimensional CBCT imaging [11]. Both of these studies suggest MARPE produces more extensive skeletal effects than conventional expanders. These results position MARPE as a promising, non-surgical intervention for adult OSA patients [2]. In contrast, Zhao et al. [6] reported that despite significant increases in maxillary width following RME, oropharyngeal airway volume remained relatively unchanged in children and adolescent patients [6]. Concurring with these observations, Cheung et al. [13] compared different expanders, including the Hyrax, Hybrid-Hyrax, and Keles Keyless expanders. Findings indicated that although all devices contributed to increased airway dimensions, the expander that yielded the most pronounced change, particularly among pre-peak growth patients, was the Hybrid-Hyrax [13].

### 3.1. Effects on Obstructive Sleep Apnea (OSA)

In 1998, Cistulli et al. [5] published one of the first studies that evaluated rapid maxillary expansion and its effects on patients with OSA. The study included 10 patients with mild-moderate OSA who underwent RME over 24 days followed by a 3–10-month follow-up. The polysomnography results after treatment showed a significant decrease in the apnea/hypopnea index (AHI) in all patients but one, as well as significant improvements in nasal respiration, daytime sleepiness, and snoring [5]. When maxillary constriction is present in OSA patients, RME has been proven to improve nasal airflow. This can lead to creating lower sub-atmospheric inspiratory pressures, therefore reducing the patients’ vulnerability to pharyngeal collapse [5].

In a similar 2022 study, Brunetto et al. [2] evaluated airway parameters in non-obese adults with OSA after using a maxillary skeletal expander (MSE) MARPE design. This expander features a jackscrew that is positioned on the posterior side, along with mini-implants with bi-cortical engagement for more effective and parallel expansion [2]. This study reported a significant reduction in AHI (65.3%), improvements in mean oxygen saturation, snoring, and overall sleep quality following treatment with the MARPE. Patients with greater pharyngeal obstructions usually will have the best clinical outcomes after palatal expansion [2]. Brunetto and colleagues also determined that posterior maxillary expansion produced greater improvements in airflow resistance, with more substantial effects on both the oropharyngeal and nasopharyngeal regions.

Supporting this, Choi et al. [14] found that nonsurgical MARPE produced acceptable long-term skeletal stability in young adults, reinforcing its utility in skeletally mature patients without the need for surgical intervention [14]. Additionally, Cantarella et al. [12] demonstrated that MARPE can achieve separation of not only the midpalatal suture but also the pterygopalatine suture, as verified through three-dimensional CBCT analysis, indicating more extensive skeletal effects than conventional expanders [12].

Collectively, these studies span nearly three decades and include both pediatric and adult patients with OSA. Each indicates that maxillary expansion produces significant improvements to sleep and airway outcomes.

### 3.2. Nasal Airway Changes in Bone-Borne and Tooth-Borne Rapid Maxillary Expansion Treatment

In the study by Kabalan et al. [15], a comparison was made between an implant-anchored bone-borne expander and a tooth-borne Hyrax expander in subjects aged 11 to 17 years old. The study evaluated changes in airway volume and minimum airway cross-sectional areas following RME treatment utilizing both CBCT and AR readings. Results indicated that there were no statistically significant correlations observed for any of the pairings (*p* > 0.05), and the results were highly variable [8]. Both tooth-borne and bone-borne appliances, as well as the control groups, showed similar variability in the results between timepoints. No significant difference was seen between treated groups and controls. The study noted that while airway volume increases were evident, the correlation between skeletal expansion and airway changes was markedly variable, indicating the multifaceted nature of these interventions [8].

Supporting this, a systematic review by Khosravi et al. [16] compared randomized controlled trials of bone-borne and tooth-borne expanders in adolescents and adults with transverse maxillary deficiency. Across eight studies involving 289 participants, the review found no significant differences between the two appliance types in terms of skeletal or dental expansion, stability, or patient-reported pain. These findings reinforce the notion that while both appliance types are effective for achieving transverse maxillary expansion, neither demonstrates consistent superiority in airway outcomes [15]. Future research should prioritize long-term, standardized trials to clarify whether specific patient subgroups could benefit more from one approach than the other.

### 3.3. Impact on Nasal Airway Volume and Resistance

Gokce et al. [17] conducted a randomized clinical trial comparing Tooth-Tissue Borne (TTB), Tooth-Borne (TB), and Bone-Borne (BB) RME appliances and their effect on the nasal airway. Patients with an age range of 12–14 years who did not reach their pubertal peak in skeletal growth were included in the study. AR was used to evaluate airway changes, which showed significant increases in nasal cavity volume across all groups. Findings indicated RME reliably enhances nasal airflow, and the choice of appliance may be a secondary consideration [16]. It was noted that the TTB group showed continued expansion during the retention phase. They also observed that each appliance consistently produced statistically significant increases in both minimal nasal cross-sectional areas (MCA) and overall nasal cavity volume [16].

The study conducted by Doruk et al. [18] looked at nasal airway resistance in growing children with maxillary constriction during RME [17]. A bonded RME appliance was used in this study, and AR was utilized to measure nasal airway resistance before treatment, during, and after treatment. Results showed that the nasal airway resistance was significantly reduced after the use of RME, and almost 60% of patients reported an improvement in their nasal breathing after treatment [5]. These findings align with the earlier work of Hartgerink et al. [19], who demonstrated that RME produced measurable reductions in nasal airway resistance using similar AR methods [19]. Together, these studies demonstrate RPE leads to improvement in nasal airway volume and resistance in similar-aged, growing patients. Benefits relating to the nasal airway were observed across different appliance types.

### 3.4. Oropharyngeal Airway Changes After Rapid Palatal Expansion

Changes in the oropharyngeal airway following RPE remain a debated topic in the literature. While RME is consistently associated with increased nasal cavity dimensions, evidence for improvements in the retropalatal and retroglossal regions is mixed. Zhao et al. [6] demonstrated that despite significant increases in maxillary width, there were no significant changes in oropharyngeal airway volume (retropalatal and retroglossal) when compared to controls using CBCT after RPE [6]. The findings of this retrospective study challenge the assumption that RME will always lead to an increase in airway dimensions. However, findings indicated that retropalatal airway volume significantly changed after expansion treatment. Zhao et al. [6] referenced additional researchers evaluated other components of the airway using AR and found RPE to be effective in widening the nasal cavities and increasing internasal volume. Additionally, a meta-analysis by Santana et al. [20] systematically reviewed the effects of RME in children with obstructive respiratory disorders. Their analysis found consistent increases in both internasal and inter-zygomatic distances as well as oropharyngeal volume following expansion. Despite these positive anatomical findings, the overall quality of evidence was rated as very low due to methodological limitations and small sample sizes [20]. These studies make evident the importance of further research in evaluating palatal expansion and its impact on oropharyngeal airway, specifically [6]. Increases in airway volume are not always paralleled by improvements in functional outcomes, indicating the need for integrated evaluation of both dimensions.

### 3.5. Comparison of RME Appliances on Airway Volume

Given the variability in RME appliance design raises the question of whether certain expanders produce greater airway improvements than others. Cheung et al. [13] conducted a randomized controlled trial to assess the short-term changes in upper airway volume after RME using three different expansion appliances [13]. The appliances used were the conventional tooth-borne Hyrax, tooth-bone-borne Hybrid-Hyrax, and Keles Keyless. The results of the expansion were evaluated via CBCT in patients from 10 to 16 years of age. They found that while all produced measurable increases in airway dimensions, the expander that was particularly effective among the pre-peak growth patients was the Hybrid-Hyrax expander compared to the conventional Hyrax. Patients who were in their CVM stages between 1 and 3 had a greater increase in total airway volume, and all three expanders showed greater change in patients with a smaller baseline airway volume [13]. Despite observed trends, comparisons between RME appliances revealed no statistically significant differences, indicating similar airway improvements regardless of appliance design.

Similarly, a randomized controlled trial conducted in Brazil also compared the Hybrid Hyrax and conventional Hyrax expanders in 42 adolescents aged 11–14 years. Unlike Cheung et al. [13], this study found statistically significant differences favoring the Hybrid Hyrax, particularly in the premolar and molar regions of the nasal cavity and maxilla [21]. In addition, the Hybrid Hyrax demonstrated fewer dental side effects, such as premolar tipping, suggesting a biomechanical advantage when skeletal changes are prioritized.

Together, these studies indicate a consistent trend toward greater skeletal and airway expansion with RME and specifically Hybrid Hyrax appliances. The strength of evidence varies, with some trials demonstrating significance and others reporting only non-significant trends.

### 3.6. Timing of RME on Nasal Airway Outcomes

Because skeletal growth influences airway adaptability, the age at which RME is implemented plays a critical role in treatment outcomes. Bicakci et al. [9] evaluated the timing and long-term effects of RME on the nasal airway. Authors found patients treated prior to their pubertal growth peak experienced greater short-term increases in nasal cavity dimensions and craniofacial skeletal changes than those who underwent expansion during or after the peak of skeletal growth [9]. Results demonstrated that patients treated before their growth peak experienced larger increases in the minimum cross-sectional area of the nasal cavity. These improvements remained more stable long-term compared with post-peak interventions [9].

Results from AR found that patients treated before the pubertal growth peak experienced greater increases in the minimum cross-sectional area of the nasal cavity. This increase remained more stable long-term compared with patients treated with RME during or after the peak.

Another systematic review and meta-analysis by Hariharan et al. [22] assessed the effectiveness of RME in the management of OSA using objective respiratory parameters. Their analysis of nine clinical studies concluded that RME was effective in reducing AHI and improving oxygen saturation across both pediatric and adult populations. The most pronounced benefits were observed in pre-pubertal patients presenting with clear maxillary deficiency. Findings indicate that age and skeletal maturity not only influence structural changes in the airway but also determine the extent to which these changes translate into measurable functional improvements in sleep-disordered breathing [22].

Santana et al. [20] also conducted a meta-analysis focused on children with obstructive respiratory disorders. Authors found RME significantly increased internasal distance and oropharyngeal volume, with improvements continuing beyond 12 months. Findings further emphasize that early intervention prior to skeletal maturation produces optimal airway benefits [20]. McNamara et al. [23] similarly concluded that RME in growing patients widens the nasal cavity base, therefore reducing airway resistance and improving respiratory patterns [23].

### 3.7. Effect of Rapid Maxillary Expansion on Nasomaxillary Structure and Sleep Disordered Breathing in Children with Obstructive Sleep Apnea

In a recent study by Pirelli et al. [4], 23 children aged 9 to 12 with malocclusions and a diagnosis of obstructive sleep apnea were evaluated via questionnaires, clinical assessments, polysomnography, and orthodontic evaluations following RME treatment. All patient cases had a successful opening of the mid-palatal suture, and results showed that all areas of the airway, including the upper airway, nasal cavity, nasopharynx, and oropharynx, and the nasal osseous width, all increased significantly [4]. All patients showed an increase in maxillary width as well as the pterygoid processes. Both 2D and 3D measurements were completed to provide results demonstrating RME is an effective mechanism for improving breathing function during sleep, supported by polysomnography outcomes and patient-reported questionnaire data.

### 3.8. Limitations

Several limitations should be acknowledged in this review. Although the initial search yielded a broad set of articles, the final number of included studies was relatively small, and many had modest sample sizes. This may limit the generalizability of findings. Second, there was notable diversity across studies in terms of patient age, skeletal maturity, appliance design, and outcome measures, including CBCT, AR, and polysomnography. This complicates direct comparisons and pooling of results. Furthermore, the overall quality of evidence varied, with several included studies being retrospective or non-randomized in design. This introduces the potential risk of bias. Non-English studies were excluded and could contribute to language bias. Finally, most studies reported relatively short-term outcomes, with a smaller number assessing the long-term stability of airway or respiratory changes following RME. Future studies with larger sample sizes, standardized outcome measures, and long-term follow-up will be essential to clarify the true functional impact of rapid maxillary expansion on airway dimensions and sleep-disordered breathing.

## 4. Discussion

The findings from the evaluated studies have provided insights into the effects of RME on upper airway volume, nasal patency, and the treatment of OSA. Although the effects of RME, both skeletal and dental, are well established, their influence on airway dimensions and respiratory function continues to stimulate active debate [23,24].

Current literature aims to summarize the effect of rapid RME on upper airway dimensions as well as breathing function in children and adolescents. The systematic reviews included evaluated changes in the upper airway while excluding those studies that focused on palatal anchorage with mini-screws or surgically assisted expansion. Non-surgical options such as maxillary expansion are increasingly recognized as valuable approaches for managing obstructive sleep apnea [25]. The results indicated that there was significant evidence that suggested RME led to improvements in nasal respiratory capability, nasal volume, as well as transverse expansion [10]. While there is significant evidence suggesting that RME leads to improvements in nasal volume, in the nasal respiratory capability, and in transverse enlargement, inconsistencies and disagreements, particularly regarding changes in the oropharynx, result in reservations about the consistency of these effects and whether they directly translate to improved breathing function [10]. The diversity in imaging modalities, study designs, and patient maturational stages provides an explanation of the variability in airway and functional outcomes of RME.

In a systematic review by Baratieri et al. [26], long-term reports of rapid maxillary expansion and its effect on airway dimension and function were evaluated [26]. The results of the eight studies selected for evaluation showed that, although there is variability in patient- and protocol-dependent factors, there is still evidence suggesting that treatment with RME can improve nasal breathing in growing children [26]. The overall timing of the interventions was noted to be the most important variable for predicting RME orthopedic outcomes.

Interpretation of these findings suggests the observed variability may be influenced by both biological and methodological factors. Younger, pre-peak patients generally exhibit greater adaptability of skeletal structures, producing more consistent airway enlargement. In contrast, adults often require greater skeletal support to overcome sutural resistance. This may explain why MARPE demonstrates superior outcomes in this group [27]. Additionally, the choice of imaging method likely contributes to differences in reported airway changes, as CBCT captures static skeletal volume and AR reflects functional resistance. This methodological diversity demonstrates the need for standardized evaluation protocols.

Collectively, these studies highlight the intricacy of how maxillary palatal expansion can impact the airway. Although there is clear evidence that can support an increase in nasal airway volume in multiple studies, the functional respiratory benefits are not as consistent. Factors such as skeletal maturity, individual anatomical variation, and specific appliance design invariably influence the results, as seen in the multiple studies evaluated. As the research landscape evolves, it is essential to focus on a more personalized, patient-centric approach to treatment planning for patients with maxillary constriction and sleep-disordered breathing.

In addition to these broader systematic reviews, individual clinical studies have evaluated the effects of RME on airway dimensions and airway outcomes. Table 1 summarizes key studies across different patient populations, appliances, and measurement methods. These investigations provide insight into how different types of RME appliances impact specific airway regions and functional respiratory outcomes. Authors consistently report increased nasal volume and reduced resistance, but findings for oropharyngeal changes and objective sleep metrics such as AHI remain variable.

It was consistently seen across the included literature that RME produces considerable increases in airway volume. Multiple randomized clinical trials reported that the Hybrid-Hyrax expander yielded the greatest expansion of upper airway volume, especially in pre-peak growth patients [13,20]. Additional reports documented significant increases in nasal airway volume regardless of the appliance used, finding no noteworthy difference among tooth-borne, tooth-tissue-borne, or bone-borne designs. These findings indicate that although RME can expand the airway, the specific design or type of expander used may not be the determining factor in the significance of these changes [15]. Table 2 summarizes representative findings across adolescent and adult populations. The table shows that while measurable increases in airway volume are generally achieved regardless of appliance type, differences in outcomes across designs often fail to reach statistical significance.

Several studies raised questions regarding the functional significance of the volumetric gains associated with RME, especially with the oropharyngeal airway. Zhao et al. [6] found that there were no significant changes in the oropharyngeal airway volume following RPE, even though there was a measurable increase in molar-to-molar width [6]. Likewise, Kabalan et al. [8] illustrated considerable variability in the correlations between skeletal expansion and nasal airway changes. Ultimately, the study concluded that no significant relationship exists between these factors [8]. These results are more consistent with earlier research, which suggests that although RME can lead to significant changes in the anatomy, these changes do not always lead to improvements in respiratory function.

Findings determined that MARPE significantly improved respiratory parameters in non-obese adult patients diagnosed with OSA. Considerable improvements in AHI were reported, as well as the duration of snoring each night and oxygen saturation. This finding is particularly meaningful, as suture fusion in adults can make skeletal expansion more challenging [2]. This suggests that even in skeletally mature patients who provide more limitations on anatomical adaptation, the use of a MARPE can offer significant respiratory benefits. There was some inconsistency in the outcomes seen in adults compared to adolescents, which may be attributable to age-related biological differences and the potential for pre-existing airway restrictions in OSA patients, making even the smallest improvement in anatomical structures clinically significant [4].

The differences among these studies demonstrate critical factors that can influence the effectiveness of RME on airway expansion. First, it is essential to consider not only the age of the patient, but also the skeletal maturity, as it plays a pivotal role in younger patients, potentially leading to more adaptive responses than adults. Second, there are clear variations in the research and methodologies when evaluating airways, such as utilizing either CBCT or AR. These methods could account for differences in reported outcomes. Lastly, the length of follow-up directly impacts the conclusions that can be drawn. Results observed soon after expansion may capture immediate volumetric gains, whereas extended follow-ups better reveal whether airway improvements are maintained and clinically relevant [7].

Many studies have indicated the benefit of palatal expansion on airway volume; however, the improvements in respiratory function specifically are still considered questionable. The varying functional outcomes reported in studies suggest that additional factors such as soft tissue adaptation, neuromuscular control, and individual anatomical variations may mediate the relationship between skeletal expansion and breathing efficiency. CBCT offers reproducible 3D airway volume measurements, while AR has been shown to provide reliable estimates of nasal airway resistance and cross-sectional area. However, CBCT does not capture dynamic soft tissue or breathing function, and AR results may be influenced by patient compliance and examiner technique. Their findings should be interpreted in conjunction with functional measures when assessing the true clinical significance of rapid maxillary expansion on airway outcomes. It is essential to note that even in the studies that reported minimal improvements in respiratory function, there were still clinical parameters that showed notable differences and improvements in the airway.

In summary, while RME can reliably produce quantifiable increases in airway volume, the clinical significance of these results should be further investigated. The most compelling evidence for functional respiratory improvements is found in studies utilizing the MARPE appliance in non-obese adult patients who are diagnosed with OSA. Furthermore, traditional RME in younger populations has elicited significant airway benefits.

Future studies should place an emphasis on more long-term follow-ups that include a comprehensive measurement of respiratory function. It is critical to include comparative studies between various methods of expansion via RME or MARPE to better understand their individual roles in the management of airways.

## 5. Conclusions

This review demonstrates that rapid maxillary expansion consistently produces measurable skeletal changes and increases in airway volume, particularly within the nasal cavity. These effects are most stable when treatment is carried out in younger, pre-pubertal patients, while outcomes in adults are less predictable but may be enhanced with mini-implant-assisted techniques. Despite reliable volumetric gains, the translation of these changes into long-term improvements in respiratory function remains inconsistent, reflecting the influence of patient age, skeletal maturity, appliance design, and variability in measurement methods.

RME can serve as a valuable adjunctive therapy for sleep-disordered breathing, but it requires careful case selection and appropriate expectations [28]. Orthodontists are uniquely positioned to identify maxillary constriction and its impact on airway health and can integrate airway considerations into treatment planning [29,30]. To advance this field, future studies need standardized evaluation protocols, integration of patient-reported outcomes, and long-term follow-up to determine the clinical relevance of airway changes. RME offers potential benefits for improving dentofacial development and general health.

## Figures and Tables

**Table 1:** Summary of Clinical Studies on Maxillary Expansion and Airway Outcomes.

Study	Population	RME Type	Measurement Method	Key Airway Outcomes	OSA Outcome
Brunetto et al. [2]	Adults with OSA	MARPE	CBCT, Polysomnography	Increased airway volume	Decreased AHI by 65.3%
Zhao et al. [6]	Children/Teens	RME	CBCT	Increase in maxillary width; no significant oropharyngeal change	N/A
Cheung et al. [13]	Pre-peak growth	Hyrax, Hybrid-Hyrax, Keles Keyless	CBCT	All devices increased airway dimensions; Hybrid-Hyrax showed greatest effect	N/A
Doruk et al. [18]	Children	Bonded RME	AR	Reduced nasal airway resistance; 60% reported improved nasal breathing	Subjective Improvement
Kabalan et al. [8]	Adolescents (11–17 years)	Bone vs. Tooth-Borne	CBCT, AR	Variable airway volume increases; no significant difference between groups	N/A
Bicakci et al. [9]	Pre- vs. Post-peak growth	Tooth-Borne RME	AR	Greater increase in minimum cross-sectional area and long-term stability when treated prior to pubertal peak	N/A
Pirelli et al. [4]	Children (9–12 years) with OSA	Tooth-Borne RME	CBCT, Polysomnography	Significant increase in upper airway, nasal, nasopharyngeal and oropharyngeal dimensions	Reduction in AHI and improved sleep quality scores
Cantarella et al. [12]	Young Adults/Adults	MARPE	CBCT	Separation of midpalatal and pterygopalatine sutures with increased nasal volume	N/A
Santana et al. [20]	Children with OSA	RME	CBCT, AR	Increase in internasal distance and oropharyngeal volume maintained after 12 months	Reduction in AHI and improvement in oxygen saturation
Harihara et al. [22]	Children and Adults with OSA	RME/MARPE	CBCT, Polysomnography	Structural and functional benefits most pronounced in pre-pubertal patients	Reduction in AHI and improvement in oxygen saturation

Studies evaluating nasal and oropharyngeal airway effects and OSA outcomes across different patient populations and expansion protocols.

**Table 2:** Effects of Expander Type on Airway Dimensions.

Study	Population	Appliance Type	Nasal Volume Change	Oropharyngeal Volume Change	Key Outcome
Cheung et al. [13]	Pre-peak adolescents	Tooth-Borne (Hyrax)	+2.8 ± 1.0 (Significant within group; NS between groups)	+0.5 ± 0.7 (NS)	Most effective in early growth stages; general airway gains seen across all devices
Cheung et al. [13]	Pre-peak adolescents	Hybrid-Hyrax	+4.6 ± 1.2 (Significant within group; NS between groups)	+1.1 ± 0.9 (NS)	Largest total airway volume increase among groups
Brunetto et al. [2]	Adults with OSA	MARPE	Significant increase (>+5 cm^3^)	Significant increase (>+3 cm^3^)	Also showed 65.3% reduction in AHI and better sleep parameters
Kabalan et al. [8]	Adolescents (11–17 years)	Bone-Borne vs. Tooth-Borne	Variable; NS difference	Highly variable; no consistent correlation	Similar effects between appliance types; no superior design found
Bicakci et al. [10]	Pre- vs. Post-peak children	Tooth-Borne RME	Significant increase in MCA prior to pubertal peak	Not evaluated	Early treatment produced greater and more stable nasal airway improvement
Doruk et al. [18]	Children (7–13 years)	Bonded RME	Nasal airway resistance reduced by ~60%	Not evaluated	Majority reported subjective breathing improvement post-treatment
Santana et al. [20]	Children with OSA	Conventional RME	Significant increase in internasal distance and volume	Significant increase in oropharyngeal volume	Meta-analysis showed persistent airway enlargement beyond 12 months

Summary of airway changes reported across various expander types in adolescent and adult populations. Data demonstrates the variability in airway outcomes by appliance design and age groups.

## Data Availability

The original contributions presented in this study are included in the article. Further inquiries can be directed to the corresponding author.

## Abbreviations

The following abbreviations are used in this manuscript:

OSA	Obstructive Sleep Apnea
RME	Rapid Maxillary Expansion
MARPE	Mini-Implant Assisted Rapid Maxillary Expansion
MPE	Maxillary Palatal Expansion
CBCT	Cone-Beam Computed Tomography
AR	Acoustic Rhinometry
AHI	Apnea–Hypopnea Index
MSE	Maxillary Skeletal Expander
MCA	Minimal Nasal Cross-Sectional Areas
